# Stunting Super App as an Effort Toward Stunting Management in Indonesia: Delphi and Pilot Study

**DOI:** 10.2196/54862

**Published:** 2024-12-17

**Authors:** Kadek Ayu Erika, Nur Fadilah, Aulia Insani Latif, Nurhikmawaty Hasbiah, Aidah Juliaty, Harun Achmad, Anugrayani Bustamin

**Affiliations:** 1 Faculty of Nursing, Hasanuddin University, Jl. Perintis Kemerdekaan Kampus Tamalanrea Km.10, Makassar, 90245, Indonesia, 62 81342129855; 2 Faculty of Medicine, Hasanuddin University, Makassar, Indonesia; 3 Faculty of Dentistry, Hasanuddin University, Makassar, Indonesia; 4 Faculty of Engineering, Hasanuddin University, Makassar, Indonesia

**Keywords:** stunting, stunting prevention, mobile app

## Abstract

**Background:**

Currently, 30 million children are experiencing acute malnutrition, and 8 million children are severely underweight.

**Objective:**

This study aimed to develop a stunting super app, a one-stop app designed to prevent and manage stunting in Indonesia.

**Methods:**

This study consisted of three stages. Stage 1 used a 3-round Delphi study involving 12 experts. In stage 2, 4 experts and a parent of children with stunted growth created an Android app containing stunting educational materials. In stage 3, a pilot study involving a control group was conducted to evaluate parents’ knowledge about stunting prevention through the app and standard interventions.

**Results:**

In the Delphi study, 11 consensus statements were extracted; arranged in three major themes, including maternal health education, child health education, and environmental education; and applied in the form of the Sistem Evaluasi Kesehatan Anak Tumbuh Ideal (SEHATI) app. This app was assessed using a content validity index, with a cumulative agreement of ≥80% among the 5 individuals. The pilot study showed an increase in the knowledge of mothers of toddlers with stunted growth before and after the educational intervention (*P*=.001).

**Conclusions:**

The SEHATI app provides educational content on stunting prevention that can increase the knowledge of mothers of toddlers with stunted growth.

## Introduction

Stunting is an effect of malnutrition characterized by suboptimal physical growth caused by malnutrition in the first 1000 days of life, which can affect children’s development and cognitive abilities [1]. Currently, >30 million children in 15 countries (ie, Afghanistan, Burkina Faso, Chad, Democratic Republic of the Congo, Ethiopia, Haiti, Kenya, Madagascar, Mali, Niger, Nigeria, Somalia, South Sudan, Sudan, and Yemen) are experiencing acute malnutrition, with 8 million of them being severely underweight [2]. In Indonesia, the Basic Health Research data show that the prevalence of stunting in toddlers in 2018 reached 30.8% [3]. Further, based on the Indonesian Toddler Nutrition Status Survey, the prevalence of stunting in 2021 was 24.4% (5.33 million children) [4]. This proves that the prevalence of stunting in Indonesia is still a public health issue because the figure exceeds the limit set by the World Health Organization, which is <20% [4][Bibr R4]. The prevalence of stunting is increasing, owing to the lack of knowledge on the importance of meeting a balanced diet for toddlers during their golden age; the lack of awareness of primary caregivers, particularly mothers, in monitoring their food; and poor public knowledge on the early prevention of stunting [5]. Early stunting prevention can be started by increasing the understanding and role of the community in stunting prevention and increasing early detection programs in toddlers, which are expected to directly reduce this issue [6,7]. If inappropriately managed, stunting can lead to short- and long-term risks, such as decreased cognitive, motor, and language development, which will affect school performance, learning capacity, and other child potentials [8]. This can also incur high health expenditures and more frequent requirements for childcare, which will ultimately increase morbidity and mortality [9,10].

Currently, governments have implemented various national programs to ensure the health of mothers and children, including (1) specific intervention activities for giving iron tablets as a supplement to adolescent girls, brides-to-be, and pregnant women; (2) the promotion of exclusive breastfeeding and breastfeeding assistance; (3) classes for pregnant women; (4) information on a balanced diet; and (5) the Healthy Living Community Movement, which consists of increasing physical activity, vegetable and fruit consumption, and early disease detection [11]. At present, the prevalence of stunting in Indonesia is still very high and thus must be addressed through more innovative and creative interventions [12]. Currently, the most widely offered intervention is the use of IT, which offers easier access to health information anytime and anywhere.

The use of digital technology for health has become a popular practice when compared with other ways [13]. This is supported by the increasing use of mobile phones, as evidenced by global mobile user data reaching more than two-thirds (67.1%) of the world’s population [14]. A form of technology usage in the health sector is the use of a smartphone app, which is commonly presented as a mobile health or eHealth app and can be further developed into a super app. A super app is a holistic app concept that has recently gained popularity, particularly in transport and other services offered by start-up companies such as Gojek, Grab, and Shopee. However, its use in the health sector remains unexplored. eHealth, or a super app, uses the interactions among medical informatics that are expected to reduce the prevalence of stunting in Indonesia, by referring to health services and information delivered or improved through technology [15]. In addition, assessment through this app is expected to be able to detect stunting early, monitor the condition of toddlers with stunted growth easily, and bridge stunting education to parents.

Preventing stunting is important to ensure the growth and physical development of children [16]. Having an app that aims to prevent stunting can improve the parents’ and the community’s knowledge about nutrition [17]. It can also provide easily accessible and relevant information and easily monitor the growth and development of children.

Several studies have evaluated the role of eHealth in stunting prevention. For example, Permana et al [18] took advantage of the use of “Nutrimo” or nutrition monitoring. Rianti et al [19] developed an app to prevent babies from being born underweight, which focuses on the health of pregnant women during pregnancy, particularly to comply with taking iron tablets. Kasjono and Suryani [20] developed Gerakan Anti Stunting (GASING, antistunting movement), an app for the delivery of information on a balanced diet and the promotion of clean and healthy living behaviors. Utario and Sutriyanti [21] developed an offline app for stunting management for Integrated Service Post task forces. However, current apps are neither comprehensive nor integrated into one platform and do not focus on stunting prevention. Therefore, this study is interested in developing a stunting super app, a one-stop app that can help prevent and handle malnutrition and thereby facilitate the eradication of stunting in Indonesia.

## Methods

### Study Design

#### Overview

This study consisted of three stages. In the first stage, content development related to stunting prevention education was performed using the Delphi study. The Delphi study is a method used to create context and collect opinions from experts on a particular topic [22]. In the second stage, the app was developed, which was later named Sistem Evaluasi Kesehatan Anak Tumbuh Ideal (SEHATI). In the final stage, a knowledge assessment was performed before and after the health education using this app with standard educational media.

#### Stage 1: Content Development by Experts

In stage 1, a classic Delphi study and web-based Delphi study were performed in 3 rounds for content development. This study involved 12 experts who met the inclusion criteria: expert educational backgrounds in medicine, nursing, nutrition, or physiotherapy who had experience with stunting for >2 years in Indonesia or parents who had children with stunting. In the first round of the Delphi study (first questionnaire), a classical Delphi study was performed by distributing a questionnaire with open-ended questions. Participants were asked, “What do you think can be done to prevent stunting?” The questionnaire was sent as a Google Form via WhatsApp version 2.24.23.78 (Meta Platforms). The answers obtained on the first questionnaire were collected and analyzed.

In the second Delphi round (second questionnaire), the analyzed answers were then sent back to the experts and scored using a 4-point Likert scale: highly relevant, relevant, irrelevant, and highly irrelevant. The answers were analyzed with a cumulative agreement indicator of ≥80%. In the third Delphi round (third questionnaire), answers that obtained a cumulative approval of ≥80% were returned for final validation, and expert approval was achieved by assessing the appropriateness of each answer component based on the Likert scale. These stages generated the final consensus.

#### Stage 2: App Development by IT Experts

IT experts developed the app based on the consensus obtained in the first stage. The content validity index (CVI) test involved 5 experts, who were not the experts employed in the previous stage, with the CVI standard of ≥80%. These participants were experts in nursing, medicine, IT, or language and a parent who had toddlers with stunted growth.

The app was developed beginning with understanding the needs of the target audience through stakeholder meetings with health care professionals and potential users. We defined the app’s core features, such as growth monitoring, educational content, and consultation tools. We then chose Flutter 3.13.0 (Google) for its responsive and visually appealing user interface, and then we moved on to designing and prototyping using human-computer interaction principles like accessibility, usability, and user experience. Wireframes were created using Figma 24.33.0 (Figma) to outline the app’s layout, and detailed designs were developed focusing on user experience and navigation. An interactive prototype to simulate how the app will work and gather feedback from stakeholders to make improvements was then built. The app was then developed using Flutter and Dart 3.3.0 (Google). Flutter allowed for a responsive and attractive user interface, while Dart was the programming language used for app development. Firebase was set up for back-end services, including data management and real-time updates, and Firebase Authentication was implemented to manage user log-ins and security. We continuously tested the app to find and fix any issues. This included unit tests for individual parts and integration tests for how different features worked together. The app was prepared for launch by submitting it to the Google Play Store, ensuring it met all guidelines. After the launch, we continued to support the app by fixing bugs, adding new features, and improving performance based on user feedback.

#### Stage 3: Stunting Super App Evaluation

A pilot study was conducted to evaluate changes in parents’ knowledge about stunting prevention after intervention using SEHATI. Knowledge assessment took place using the contents developed in stage 1. In this stage, the pilot study involved 30 participants selected based on the inclusion and exclusion criteria. Inclusion criteria were participants who could use a mobile phone and mothers who have children younger than 5 years. Meanwhile, the exclusion criteria were participants who use iOS software on their mobile phones. In the pilot study, the recommended sample size was at least 12 participants [23]. Changes in knowledge of stunting prevention were assessed, and changes attributed to the use of SEHATI and other educational media were compared. Parents (1) with toddlers aged <2 years (2) who possessed and could operate a smartphone (3) and were willing to participate in the study were enrolled.

The app was given once to respondents, starting with an explanation of the manual book by the research team. After the app was installed and respondents could use the SEHATI app, respondents were given educational interventions to prevent stunting through the app. Respondents could access the app anytime and anywhere because it could be used offline, so it was also suitable for use in rural areas that have limited internet access.

### Data Analysis and Statistical Test

Descriptive statistical tests were used to assess the characteristics of the respondents. The results are displayed as mean, SD, and percentage. The results obtained from the consensus of the experts were integrated into an app, and the CVI test was taken with expert judgments. The CVI indicator was considered valid if the value was ≥0.78. Furthermore, bivariate analysis was performed to assess the increase in knowledge in the group using the nonparametric Wilcoxon test and Mann-Whitney test. All data were analyzed using IBM SPSS Statistics for Microsoft Windows version 21 (IBM Corp).

### Ethical Considerations

This study received Health Research Ethics Commission approval (#126/UN4.6.4.5.31/PP36/2023). Before the study, all experts received a consent form, which explained the purpose, procedures, benefits, confidentiality, risks of the research, and the right to refuse or freely withdraw participation in the study. All expert data were anonymized. Participants were given compensation in the form of internet data vouchers (US $18.84).

## Results

### Delphi Study

The educational theme for mothers consisted of 4 contents with a cumulative agreement presentation of ≥80%, including preparing prepregnancy nutrition, meeting nutritional needs during pregnancy, routine pregnancy checks, and regular intake of vitamins during pregnancy. Meanwhile, the educational theme for children that met the cumulative agreement presentation of ≥80% had 4 contents, namely, exclusive breastfeeding, provision of complementary feeding, routine monitoring of children’s growth and development, and complete immunizations. Meanwhile, the theme for environmental health education included 3 contents, with a cumulative agreement presentation of ≥80%, including environmental cleanliness, parenting, and socioeconomic support.

In stage 1, the Delphi study was conducted to develop a stunting prevention educational app involving 12 experts, and the 11 contents identified were grouped into three major themes, namely, educational content for mothers, children, and the environment (Table 1).

**Table:** **Table 1**. Result of stage I of the Delphi study: educational contents for stunting prevention.

Topic	Prevention components	Likert scale responses (n=12), n (%)				Cumulative agreement, n (%)
		Highly irrelevant	Irrelevant	Relevant	Highly relevant	
Maternal health educational contents						
Preparing prepregnancy nutrition	Brides prepare nutrition before pregnancy (prepregnancy) by consuming a balanced diet (carbohydrates, vegetables, animal proteins, vitamins, and minerals) and iron tablets at the age of 12‐18 years with a dose of one tablet per week. Mothers also need to take height and weight measurements to monitor BMI.	0 (0)	0 (0)	3 (25)	9 (75)	12 (100)
Meeting nutritional needs during pregnancy	Pregnant women must meet their nutritional needs by consuming macronutrients and micronutrients. a. Macronutrients Carbohydrates such as rice, cassava, corn, sweet potatoes, and others Proteins from plants such as beans, tofu, tempe, and others Proteins from animals such as meat, chicken, eggs, and milk, and others b. Micronutrients Multivitamins contained in vegetables and fruits Minerals such as calcium, folic acid, iron, and iodine	0 (0)	0 (0)	3 (25)	9 (75)	12 (100)
Routine pregnancy check	Mothers routinely undergo pregnancy checks at least six times during pregnancy, as follows: First trimester (gestational age 0‐12 weeks): 1 time Second trimester (gestational age>12‐24 weeks): 2 times Third trimester (gestational age>24‐40 weeks): 3 times	0 (0)	0 (0)	2 (17)	10 (83)	12 (100)
Routine vitamin consumption	Mothers routinely: Take folic acid tablets once a day after meals during pregnancy Take one iron tablet every day during pregnancy, for at least 90 tablets, to prevent blood deficiency (anemia) and start as early as possible	0 (0)	0 (0)	3 (25)	9 (75)	12 (100)
Children’s health educational content						
Exclusive breastfeeding	Mothers give breast milk exclusively to newborns up to 6 months of age.	0 (0)	0 (0)	3 (25)	9 (75)	12 (100)
Complementary feeding	Mothers feed complementary foods that are adjusted to the age (month) of the child	0 (0)	1 (8)	1 (8)	10 (84)	11 (93)
Routinely monitor children’s growth and development	Mothers routinely monitor the growth and development of children by weighing and measuring the length or height at each visit to the Integrated Service Post with the following frequency: Monthly visit for toddlers aged 0‐12 months Every 3 months for toddlers aged 12‐24 months Every 6 months for toddlers aged 24‐72 months Every 3 months for head circumference measurement at the age of 0‐12 months Every 6 months for head circumference measurement at the age of 18‐72 months	0 (0)	1 (8)	1 (8)	10 (84)	11 (93)
Complete immunization, vitamin A, and deworming	Mothers routinely take their children for complete immunization, vitamin A supplementation, and deworming with the following schedule: From birth to 24 hours old: hepatitis B, BCG^a^, and polio drops 1 1 month old: BCG and polio drops 1 2 months old: DPT-HB-Hib^b^ 1 and polio drops 2 3 months old: DPT-HB-Hib 2 and polio drops 3 4 months old: DPT-HB-Hib 3, polio drops 4, and injectable polio vaccine 9 months old: measles-rubella 18 months old: continued measles-rubella and DPT-HB-Hib Administration of blue vitamin A capsule once at the age of 6‐11 months Administration of red vitamin A capsule once every 6 months at the age of 12‐59 months Deworming from the age of 12 months, taken twice a year or every 6 months	0 (0)	1 (7)	3 (22)	8 (71)	11 (93)
Environmental educational content						
Keeping the environment clean	Sanitation, clean latrines, stay away from cigarette smoke and wear proper clothing.	0 (0)	0 (0)	3 (25)	9 (75)	12 (100)
Parenting	Parenting plays an important role in preventing stunting. Parenting is divided into *asah, asih,* and *asuh*. *Asah* refers to the stimulation of children’s intelligence, such as the provision of educational tools that help them become smarter *Asih* refers to efforts to develop a child’s affection, spirituality, independence, and need for a sense of security and comfort *Asuh* encompasses physical and biological needs, including nutritional requirements, immunization, personal and environmental cleanliness, medical care, and physical activity and play	0 (0)	0 (0)	3 (25)	9 (75)	12 (100)
Socioeconomic support	Parents must be aware that economic support plays an important role in helping children obtain their nutritional needs. One of the things that can prevent malnutrition is to save money during pregnancy, locally known as *Tabungan Ibu Bersalin* (*Tabulin*). It can be started at the beginning of pregnancy to meet maternity needs and the initial needs of the baby at birth.	0 (0)	1 (7)	3 (29)	8 (64)	11 (93)

a BCG: Bacillus Calmette-Guérin.

b DPT-HB-Hib: diptheria, pertussis, tetanus, hepatitis B, *Haemophilus influenzae* type b pediatric unspecified.

### Stunting Super App Development: Content Validity

In the second stage, the validity of the super app contents was evaluated. The contents were assessed by 4 experts from various disciplines (nursing, medicine, IT, and language) and a parent of children with stunted growth (Table 2).

**Table 2.** Quantitative evaluation of SEHATI^a^ app contents.

Assessment items	Likert scale responses (n=5), n (%)				Cumulative agreement, n (%)
	Highly irrelevant	Irrelevant	Relevant	Highly relevant
Simple design for easy use	0 (0)	0 (0)	1 (20)	4 (80)	5 (100)
Uses language and sentences that are easy to understand	0 (0)	0 (0)	2 (40)	3 (60)	5 (100)
Provides features based on user categories	0 (0)	0 (0)	1 (20)	4 (80)	5 (100)
Combines images with text	0 (0)	0 (0)	0 (0)	5 (100)	5 (100)
Avoids distracting elements (such as excessive accessories or styles)	0 (0)	0 (0)	2 (40)	3 (60)	5 (100)
Users cannot edit the app’s contents	0 (0)	0 (0)	4 (80)	1 (20)	5 (100)
Provides structured information for easy comprehension	0 (0)	0 (0)	3 (60)	2 (40)	5 (100)
Users can adjust notification sounds (alarm notifications)	0 (0)	0 (0)	1 (20)	4 (80)	5 (100)
Users can set alarms	0 (0)	0 (0)	3 (60)	2 (40)	5 (100)
Uses multiple-choice mode	0 (0)	0 (0)	1 (20)	4 (80)	5 (100)
Users can download the app through WhatsApp, Shareit, Bluetooth, or email	0 (0)	0 (0)	2 (40)	3 (60)	5 (100)
Users can take pictures	0 (0)	0 (0)	3 (60)	2 (40)	5 (100)
The app does not exceed 30 MB in size	0 (0)	1 (20)	2 (40)	2 (40)	4 (80)

a SEHATI: Sistem Evaluasi Kesehatan Anak Tumbuh Ideal.

There were 13 scoring items using 4 Likert scales (highly irrelevant, irrelevant, relevant, and highly relevant). The validity limit of the app contents was set to a cumulative agreement of ≥80%, and if the evaluation value was <80%, then adjustments were made to the app content. All items in the CVI test met the cumulative agreement presentation (≥80%). In addition, the results of the qualitative evaluation of SEHATI are shown in Table 3.

**Table 3.** Qualitative evaluation of SEHATI^a^ content.

Reviewer field	Comments of the reviewers
Nursing	*This app has provided educational needs from every critical stage for stunting prevention starting from before pregnancy in mothers until 1000 days after giving birth. In the future, there needs to be regular content updates and addition of information needed by users according to the latest developments in child health issues.* [Reviewer Ar]
Medicine	*The app is very good; however, it is necessary to add information for adolescent girls and pregnant women and monitor growth and development based on BW/BH, BMI/A, MUAC, and HC.* [Reviewer Aj]
IT	*The interface of the system is already decent and has met the needs of the users. It needs to consider space and memory upgrade to anticipate increasing users. *[Reviewer U]
Indonesian linguist	*This app benefits parents to avoid stunting. Its clear and communicative language makes this app easy to use. The design and important information regarding the health of the mother, child, and the environment are displayed very well. This app also has a consultation feature, which makes it easier for users to consult their health if they experience symptoms. Hopefully, it can be useful for the public given its simple operation and economical use.* [Reviewer M]
Mother of toddlers with stunted growth	*This app is good enough because it covers all stages of development starting from the womb, after birth, and subsequent development. Even so, several components must be added such as myths and facts related to pregnancy and children, foods to be avoided, dos and don’ts, and traditions that may ruin child development.* [Reviewer R]

a SEHATI: Sistem Evaluasi Kesehatan Anak Tumbuh Ideal.

The results showed that content must be updated regularly according to the development of the latest child health issues (nursing). Also, additional information is necessary for adolescent girls and pregnant women and for growth and development monitoring (medicine), and space and storage upgrades are required if the number of users increases (IT). The app language was clear, communicative, and economical in its use (linguist); provided consultation features; and covered all stages of development. However, some components must be added, including myths and facts about pregnancy, childhood, and inappropriate traditions (mother; Table 3).

**Figure 1.**
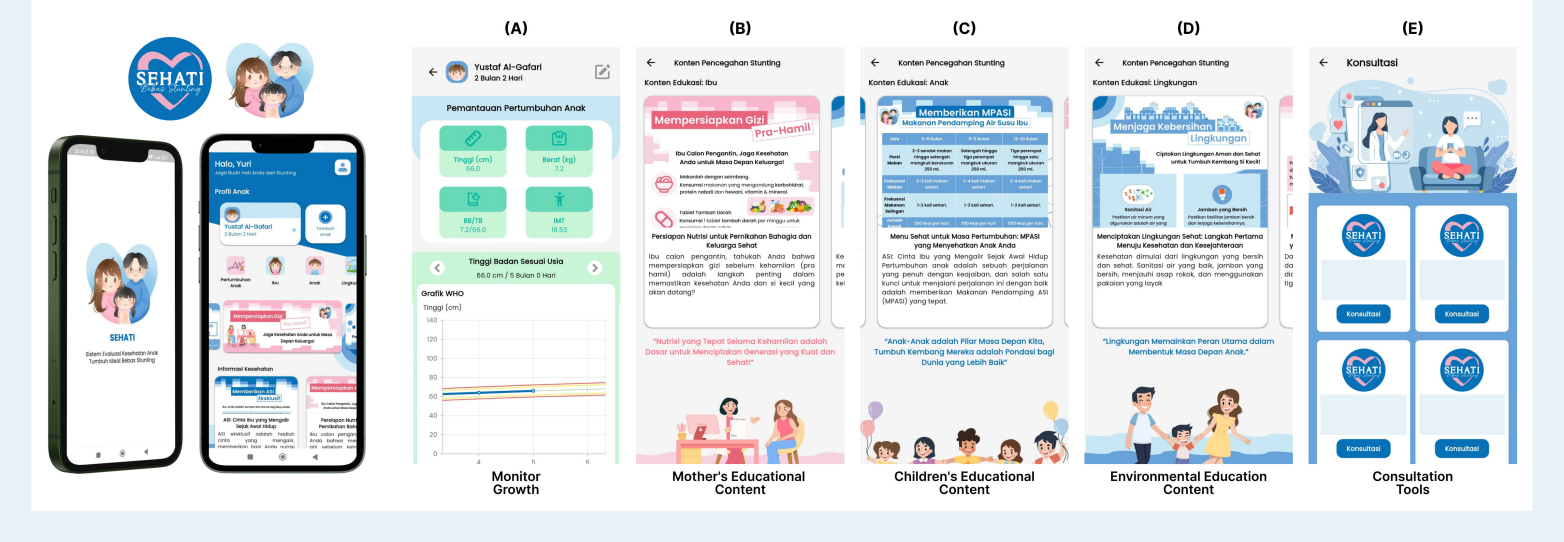
Sistem Evaluasi Kesehatan Anak Tumbuh Ideal (SEHATI) app features. SEHATI is an app designed to simplify child growth monitoring and includes stunting information. Parents can track their children‘s growth and ensure their well-being with an easy-to-use interface. (A) Track and display a child's growth through charts: weight-for-age, length/height-for-age, weight-for-length/height, and BMI-for-age. (B) Informational resources that focus on maternal health to guide mothers in making the right health decisions. (C) Learning materials to support a child’s development and improve health awareness. (D) Information on environmental factors that have an impact on children’s growth. (E) Users can consult with health experts for personalized advice regarding a child’s growth.

This app was named SEHATI (Sistem Evaluasi Kesehatan Anak Tumbuh Ideal, ie, Ideal Growth Child Health Evaluation System), which means a comprehensive, one-stop health intervention app for stunting prevention and management. The features of SEHATI are summarized in Figure 1.

Based on the results of the observations and interviews with researchers, the experience of users of this app was good. Respondents were happy because the app contained health information on stunting prevention that could be accessed easily at any time. This app was helpful for mothers in monitoring child growth, and because the app was equipped with growth charts, data could be input by mothers themselves every month. In addition, there was a consultation feature that was helpful for mothers in dealing with their children’s health.

### Pilot Study: Increasing the Knowledge of Stunting Through SEHATI

This stage was conducted to track changes in the knowledge of mothers of toddlers with stunted growth regarding stunting prevention. A total of 30 mothers who had babies or toddlers and could operate smartphones and the SEHATI app received educational intervention. Their knowledge was evaluated twice: once before education (pretest) and once after education (posttest). The average age of mothers was 29 years and the average age of the children was 6 months. Most participants had a bachelor’s degree (n=20, 66.7%), with the dominant occupation being a housewife (n=20, 66.7%; Table 4).

**Table 4.** Characteristics of the respondents by age, education, and occupation.

Characteristics of respondents	Values	
Age of mothers (years; n=30)		
Mean (SD)	29 (5.240)	
Min-max	17‐39	
Age of children (months; n=30)		
Mean (SD)	6 (7.617)	
Min-max	1‐38	
Education (n=30), n (%)		
Primary school	1 (3)	
Junior high school	2 (7)	
Senior high school	7 (23)	
Undergraduate degree	20 (67)	
Occupation (n=30), n (%)		
Housewife	20 (67)	
Government employee	9 (30)	
Self-employed	1 (3)	

The statistical test used to determine the difference between pretests and posttests was a 2-tailed *t* test because the data was normally distributed. The results showed a significant difference after participants received stunting prevention education using the SEHATI app (*P*=.001), which means there was an increase in maternal knowledge after participating in education with the app (Table 5).

**Table 5.** Differences in knowledge before and after stunting prevention education.

	Mean test score (SD)^a^		SE	*P* value^b^
Scores (n=30)				.001
Pretest	14.63 (1.326)		0.242	
Posttest	16.20 (1.495)		0.273	

a The number of pretest and posttest questions was 20.

b A paired sample, 2-tailed *t* test was done to determine significance.

## Discussion

### Preparation of Stunting Prevention Education Contents

Initially, the Delphi technique was used in a series of studies by the RAND Corporation in the 1950s [24]. It is one of the methods that the group used to survey and gather the opinions of experts on a particular topic [25]. In this study, a combination of classic and web-based Delphi techniques were used, which began with open questions to experts, and the study was conducted in three rounds. The classic Delphi used an open-ended questionnaire administered in three rounds, whereas the web-based Delphi used Google Forms distributed through WhatsApp [26]. The Delphi study was conducted in 3 rounds as adapted from Abrar et al [27].

The preparation of the app’s contents involved 12 experts, who were selected based on education level, work experience, and stunting training certificates, and they extracted three major themes, namely, maternal, child, and environmental health educational content. In addition [28], the authors involved parents who had experience in caring for toddlers with stunting, provided information about caring for toddlers with stunted growth, and gained knowledge on preventing children from experiencing the adverse effects of stunting again [29].

A total of 11 themes on stunting prevention were identified in the Delphi study. This is in line with the component of needs regarding stunting prevention in terms of maternal health in the study by Ekayanthi and Suryani [30]. In their study, the nutrition and health of pregnant women were improved by meeting the requirement for macronutrients and micronutrients, giving nutritional supplements (iron tablets), and regular monitoring of the health of pregnant women.

Stunting prevention based on children’s health is carried out by good parenting, exclusive breastfeeding, intake of appropriate complementary foods, immunization, provision of a psychosocial stimulus to children, and basic health care [31]. Environmental education also contributes to stunting prevention by practicing clean and healthy living behavior, increasing access to clean water and sanitation facilities, and maintaining environmental cleanliness [32].

### Content Validity Evaluation of SEHATI

In the second stage, the validity of the app was tested by expert judgment. The authors chose Android as the educational medium because of its effectiveness as an information tool and its increasing use for educational media [24]. According to Putra et al [33], the use of Android-based educational media is an effective medium to increase public knowledge and attitudes toward stunting. In line with this finding, Fitriami and Galaresa [34] found that Android is a comprehensive means that helps improve maternal nutritional behavior.

In this study, all items in the CVI assessment component on the SEHATI app met the cumulative agreement percentage (≥80%), and they recommended updating the contents regularly so that mothers have easy access to the latest information regarding child development issues and stunting prevention [33]. Further, young women need additional information to prepare them for the next stage of life as mothers so that stunting can be prevented [35]. In addition, more content about pregnancy myths and facts must be considered to prevent fetal stunting in mothers believing such myths [36].

In addition, the SEHATI app’s sustainability plan is that the development team will collaborate with health stakeholders for sustainable maintenance. Funding and maintenance of the SEHATI app will be charged to stakeholder institutions. SEHATI is designed to complement existing stunting prevention programs in Indonesia through comprehensive growth monitoring features and easy access to educational materials. SEHATI also strengthens the government’s efforts to monitor and improve the nutritional status of children. The app supports existing systems by providing structured, real-time growth data that can be accessed by relevant parties for further analysis under certain conditions. SEHATI also allows users to consult directly with health experts, facilitating coordination and early intervention in high-risk cases.

### Change in the Level of Knowledge

The results showed that the participants demonstrated an increase in knowledge after using the SEHATI app. The urgency of disseminating knowledge has been stated in the Regulation of the Minister of Health of the Republic of Indonesia No. 67 of 2016, which focuses on increasing true and comprehensive knowledge regarding prevention, transmission, treatment, and a clean and healthy lifestyle [37]. The app is considered effective because it is associated with efficient use of time and energy in providing education to parents who have malnourished toddlers [29]. In addition, Android apps can be accessed by phone and by everyone, allow for multitasking, and easily notify parents or the community [34]. Regarding app use by parents, a reliable, up-to-date, and evidence-based app is crucial, particularly when using apps in the context of routine care for children [38].

### Limitation

The limitation of this study was related to the sampling approach, in which experts were chosen based on the researchers’ judgment. Thus, to prevent this bias, a purposeful sampling technique was used in the selection of expert panelists, who were clinically competent and participated voluntarily.

### Conclusions

The Delphi study resulted in 11 consensus themes for stunting prevention. The results were integrated into the SEHATI app, which was assessed using the CVI and the cumulative percentage of agreement from 4 experts in nursing, medicine, IT, language, and a parent of toddlers with stunted growth was ≥80%. SEHATI as a stunting prevention educational app contributes significantly to the increase in stunting knowledge for mothers who have toddlers with stunted growth.

## Abbreviations

CVI: content validity index
GASING: Gerakan Anti Stunting
SEHATI: Sistem Evaluasi Kesehatan Anak Tumbuh Ideal
